# DIGITAL HEALTH LITERACY AND SELF-EFFICACY IN USING E-HEALTH RESOURCES AMONG CAREGIVERS OF INDIVIDUALS WITH ADRD

**DOI:** 10.1093/geroni/igae098.0165

**Published:** 2024-12-31

**Authors:** Kyeung Mi Oh, Jung-Ah Lee

**Affiliations:** George Mason University, Fairfax, Virginia, United States; University of California Irvine, Irvine, California, United States

## Abstract

This study explored digital health literacy skills, self-efficacy in utilizing e-health resources, and self-efficacy in managing personal health, with a focus on caregivers of individuals with Alzheimer’s disease and related dementias (ADRD). Data from the Health Information National Trends Survey 2022 were analyzed, involving a sample of 96 family and unpaid caregivers of individuals with ADRD. Among these caregivers, 49.9% lacked confidence in utilizing e-health resources. While over 70% utilized certain digital health tools, such as accessing medical information and viewing medical test results, usage of health or wellness apps (57%) and wearable devices (48%) was relatively lower. Engagement in sharing health information (21%) or interacting with individuals with similar health problems (33%) on social media was low, while watching health-related videos on social media (72%) was relatively high. Telehealth (59%) and patient portal usage (87% for own health and 34% for care recipient) were moderate. Controlling for demographic characteristics, each digital health literacy skill was assessed in relation to self-efficacy in utilizing e-health resources. The skill for using health or wellness apps (AOR=111.13, 95%CI: 109.45-112.83) showed the strongest association with self-efficacy, followed by the use of patient portals (AOR=15.77, 95% CI:15.58-15.97) and accessing test results online (AOR=5.64, 95% CI: 5.59-5.69). Significant correlations were found between self-efficacy in using e-health resources and managing personal health among ADRD caregivers (r=.166, P< 0.001). The insights from this study can guide the development of tailored e-health interventions to provide enhanced support for caregivers in their crucial role.

